# Narratives of Self-Harm at School: Identifying Trajectories of Intervention in Educational Contexts

**DOI:** 10.5964/ejop.v16i1.1883

**Published:** 2020-03-03

**Authors:** Anna Gargiulo

**Affiliations:** a Department of Humanities, University of Naples Federico II, Naples, Italy; Department of Psychology and Counselling, Webster University Geneva, Geneva, Switzerland

**Keywords:** self-harm, narrative, school cultural context, gender, psychodynamic perspective

## Abstract

Recent studies have shown that self-harming behaviour is increasingly widespread among adolescents, in particular at school. However, educational institutions perceive themselves unable to cope with the phenomenon, searching for protocols and guidelines to improve its management. Considering schools as useful contexts for intercepting the young malaise, this study aims at exploring the main meanings of self-harming behaviours made within the educational cultural contexts starting from the student’s narrations, in order to understand the possible trajectories of practice. In two high schools we have collected 96 narratives of self-harm written by adolescents (mean age 14; 74% females), who have been engaged in non-suicidal self-injury once in their life. The analysis of the narratives, produced with the help of a software for the automatic qualitative analysis of texts, has allowed to identify four prevalent themes organized into three sense vectors. The findings highlighted significant gender differences in the representation of the experience of self-harm between males and females, as well as the importance of meaningful relationships developed in familiar and educational contexts, which may allow the help seeking process. The emerging of culturally-shared meanings among adolescents within the investigated contexts may allow to think about possible protocols of preventive and clinical practices in schools.

The self-harming behaviours have been defined as superficial and intentional injuries of one’s body tissue, inflicted without conscious suicidal intent and for not socially accepted reasons (Favazza, 1996; Gargiulo et al., 2014); examples are behaviours such as cutting, burning and scratching the skin.

In recent years we have seen an increase in these behaviours, particularly widespread among the school youth population (Lewis & Heath, 2015). In particular, recent studies have shown that self-harming behaviours increase in adolescence (Brown & Plener, 2017), and tend to decline in late adolescence (Plener et al., 2015), with high rates of incidence in pre-adolescence (Brown & Plener, 2017; Heath, Toste, Nedecheva, & Charlebois, 2006).

Concerning to the gender variable, great part of the literature shows that girls are more likely to implement the indicated behaviours than males (Bresin & Schoenleber, 2015; Valencia-Agudo et al., 2018), although other studies show a gender gap, less evident in prevalence (Klonsky, 2011; Sornberger et al., 2012), but linked to aetiology, trajectories and contexts (Gargiulo & Margherita, 2014; Whitlock & Rodham, 2013). Significant gender differences have been found with respect to the kind of self-harm (girls mostly resort to cutting), to the injured body parts, to the age of onset (earlier for girls), to the increase in self-harming behaviour and to the suicidal ideation in pre-adolescence (Andover et al., 2010; Sornberger et al., 2012), to the access to the request for help (Heath et al., 2010), as well as to the social dimensions of self-harm (the boys tend to self-harm in group; Whitlock et al., 2011).

A part of the literature has shown that adolescents who self-harm have difficulty in putting their sufferings into words and consider self-harm as a means of communication that allows them to share the emotions of anger and anguish and which allows families and those who surround them to realize how much they were suffering (Abrams & Gordon, 2003; Crouch & Wright, 2004; Fortune, Sinclair, & Hawton, 2008; Moyer & Nelson, 2007). In fact, it has been shown that teenagers who self-harm have a poor ability to identify, name and express their emotions (Gratz, 2007; Paivio & McCulloch, 2004) and they report the affective regulation as the most common motivation to get injured (Laye-Gindhu & Schonert-Reichl, 2005; Madge et al., 2008; Nock & Prinstein, 2004). Thus, self-harming behaviour becomes a strategy for the regulation of emotions that replaces the possibility of expressing negative emotions through language. From this point of view, self-harm has been regarded as a primitive form of communication (Farber et al., 2007), pre-symbolic state (Gregory & Mustata, 2012), difficulty in symbolizing and mentalizing affects (Haza & Keller, 2005; Rossouw & Fonagy, 2012). On the other hand, the adolescence represents a phase of development in which the instinct can physiologically exceed the capacity of representation of the adolescent, who can resort to acting out as a specific phase mechanism, in which impulsive behaviours are used to express, through the body, emotions that cannot be put into words yet (Blos, 1967).

From a psychoanalytic perspective, the self-harm has been considered as an extreme attempt to search for individuation through the contact of the body limit (Biven, 1982; Fattori, 2013). Furthermore, the construction of one’s own identity, as a structuring of a feeling of Self separated from the other, endowed with structure and boundaries, represents one of the evolutionary tasks of the adolescent process (Blos, 1967). In terms of object relations, the psychodynamic literature has shown that the conduct of self-harm can fulfil some unconscious tasks, related to the attempt to separate from the other significant, made possible only by ‘cutting away’ in a violent way the other lived as living intrusively his own body (Lemma, 2005). Thus, the object is attacked whose dependence is intolerable, denying its loss. At the same time, the pain for the loss of the bond is experienced with peaks of despair, performed and evacuated in self-destructive actions (Ruggiero, 2007).

## The Behaviours of Self-Harm at School

Starting from the first school study (Ross & Heath, 2002), much of the research aims at investigating self-harming behaviour in adolescence was carried out in schools (De Leo & Heller, 2004; Hawton et al., 2002; Madge et al., 2008; O’Connor et al., 2009), showing a prevalence of behaviour between 14% and 20% of high school students (Laye-Gindhu & Schonert-Reichl, 2005; Ross & Heath, 2002). Recent international studies show an increase in self-injurious behaviours in schools with a female prevalence (Muehlenkamp et al., 2009): in fact, 18% of teenagers who attend school report to have been severely self-harmed at least once in their lifetime (Muehlenkamp, ​​Claes, Havertape, & Plener, 2012; Swannell, Martin, Page, Hasking, & St John, 2014). In particular, in Italy, lifetime prevalence of 42% for self-harming behaviours was observed in a sample of adolescents from high schools, without substantial gender differences (Cerutti, Manca, Presaghi, & Gratz, 2011).

However, although self-harming behaviours commonly emerge in the scholastic context, where teachers and other scholastic professionals often represent the first contact, studies show that the school staff is perceived as uncertain and impotent in addressing the difficulties associated with these behaviours with students (Berger, Hasking, & Reupert, 2014). In fact, despite 74% of teachers report to have come across cases of self-harm during their working career, perceiving that the behaviour is increasing in schools, 78% of them underestimate the spread of the same at school (Best, 2004; Heath, Toste, & Bettam, 2006).

Therefore, the need to prepare some clear guidelines for the management of self-harm behaviour at school is gaining ground. Contemporary literature is questioning the issue and various protocols have been produced (Bubrick, Goodman, & Whitlock, 2010; Hasking et al., 2016; Toste & Heath, 2010), sensitive to the identification of self-harming behaviours, to the response of the school staff, to the involvement of parents, to the respect for the ethical grounds of intervention in educational institutions, as well as to the management of possible phenomena of infection of the conduct at school (Walsh, 2012). However much still needs to be done in order to intercept the specific needs of teenagers with self-harm, to identify gender differences and to prepare targeted interventions. On the other hand, the school represents the most suitable context for the prevention of self-harm (Shaffer & Gould, 2000), since it is possible to get in contact with most adolescents at risk of self-harm, achieving less stigmatizing interventions than those conducted in specialized clinical centres.

## The Elaborative Function of Narrative

Assuming a cultural and semiotic psychology perspective (Salvatore & Freda, 2011; Valsiner & Rosa, 2007), each semiotic dynamic is considered always a socially distributed and intersubjective process, as well as deeply embodied and affective (Salvatore, 2016; Salvatore & Zittoun, 2011; Valsiner & De Luca Picione, 2017). The language, as a semiotic mediator, enables to narratively construct one’s own identity and one’s experiences with others (Salvatore, 2016; Valsiner, 2001; Vygotsky, 1987). In this framework, the narration is a psychological, intersubjective and cultural process (Brockmeier, 2000; Bruner, 1990; Schafer, 1992; Spence, 1982) which allows to construct meaning (Martino, Lemmo, et al., 2019; Neimeyer, Burke, Mackay, & van Dyke Stringer, 2010) and to reconfigure temporal perspectives (Brockmeier, 2000; Martino, Gargiulo, Lemmo, & Margherita, 2019), supporting processes of transformation and elaboration of traumatic experiences (Margherita, Boursier, Gargiulo, & Nicolò, 2017; Valsiner, 2007). During their lifetime, individuals constantly re-configures the narration of their experiences contextually, within a recursive, open, intransitive cycle in mediating self-other-world (De Luca Picione, 2015; De Luca Picione & Valsiner, 2017).

In adolescence, several studies have investigated the narrative development (Habermas & de Silveira, 2008; McAdams, 1993; McLean & Breen, 2009; McLean & Mansfield, 2012), considering it as an important attempt for young people to understand oneself and an opportunity to articulate their own feelings and thoughts about the self, the Others and the events. In particular, researches on narratives from adolescents who self-harm, highlighted the presence of a magical thinking in their language to elaborate the emotions (Gregory & Mustata, 2012), and family difficulties and traumas as sources of underlying distress in young women (Abrams & Gordon, 2003).

Nowadays, the self-harm among young people is narrated commonly through virtual communities (Lewis & Seko, 2016), where the malaise is shared throughout narratives and images of injured bodies, projecting emotions into the network, and using the same virtual space as emotional regulator (Gargiulo & Margherita, 2019; Margherita & Gargiulo, 2018). The meanings emerging from these virtual cultural contexts propose the representation of the Self-cutter in oscillation between the normalization and the pathology (Gradin Franzén & Gottzén, 2011). Therefore, several clinical interventions are taking place within the virtual contexts in order to intercept the specific needs of young people with self-harm.

In this sense, it could be useful to explore also the meanings about self-harm emerging from school context, which constitutes a specific field of development of the preventive and clinical interventions. Considering the school as a cultural context (Ligorio & Pontecorvo, 2010; Margherita, 2007), in which its actors can build meanings and develop practices for the elaboration of experiences, and considering that the self-harm is a contemporary theme of clinical relevance in schools, the work aimed to explore the meanings about self-harming behaviours emerging from the educational institutions, in order to identify possible trajectories of intervention. To do this, it stars from the narratives produced by adolescents in high schools, enabling the emerging of contextual meanings from the intersection between the individuals and the environments.

## Method

From a perspective of cultural and semiotic psychology (De Luca Picione & Valsiner, 2017; Salvatore & Freda, 2011; Valsiner & Rosa, 2007), in which subjective experience is considered as a continuous process of formation through the mediation of semiotic devices (meaning, linguistic and aesthetic canons, ways of acting and so on), a narrative construction that allows to access to culturally shared meanings (Bruner, 1990), and the narration is considered as a device capable of activating possible fields of knowledge and transformation, we have chosen a qualitative approach aimed to explore the meanings using the narrative tool. The exploratory qualitative approach allows to start from data, and, through a bottom up method, it provides to identify the main themes.

### Participants

Within a larger research project on self-harm, which involved 589 students from two public high schools in Naples, 97 narratives written by adolescents, who, through a series of funnel questions, had declared that they had implemented self-harming behaviours at least once in life (72 females and 25 males, average age 14) were collected.

### Tools

A narrative task was arranged to invite to narrate an episode of self-harm (“*Tell a story in which the protagonist intentionally wounds his body. The history can be real or completely invented.”* / “*Racconta una storia in cui il protagonista ferisce intenzionalmente il suo corpo. La storia può essere reale o inventata.”*). The participants were asked to indicate whether the protagonist was an imaginary or real character, indicating in this case whether the narration concerned themselves or others.

### Procedure

After contacting twelve higher institutes in Naples, in the two which decided to collaborate, the research passed to the scrutiny of the Ethical Commissions. The research design was also approved by the Ethics Committee of the Federico II University of Naples. The meetings with the classes for the research were followed by a moment of group discussion with the students. All participants signed the informed consent form for the research. In addition, research return days open to parents and teachers have been set.

### Data Analysis

Within an explorative and qualitative methodology of research, we used a thematic analysis of narrative texts. To analyse the narratives, we used T-Lab (Lancia, 2004), a qualitative-quantitative software for texts automatic analysis. We choose to use T-Lab because, based on a comparison of different lexical profiles, it identifies dimensions of meanings, shared themes or issues present in the text under analysis, through the study of vocabulary (Bolasco, 1999). The software analyses the texts as a single set of data (Denzin & Lincoln, 1994), identifying the lexical choices and carrying out on them co-occurrence and comparative analysis. Each lexical choice, the word, has a meaning in the ‘relationship’ with the other words, therefore, within a discursive dynamic it is observed how much that word presents itself recursively in the relationship with the others (co-occurrence). In this way, the frequency of lexical choices informs us of the semiotic process of meaning construction, allowing to understand how a person constructs meanings of his/her experience.

The narratives as documents were codified following the variables considered, and all the documents were merged into a single body of text. The variables considered were: sex of participant (female; male); protagonist of the narrative (myself; real other; imaginary protagonist). The documents analysed are equal in size of textual corpus for the balancing of statistical weight. We carried out a preliminary treatment of the text (Table 1).

**Table 1:** Preliminary Organization of the Text

Type of treatment	Explication
Lemmatization	The forms of the verbs are brought back to their present infinitive forms, the nouns and adjectives to their singular masculine form, articulated prepositions to their article-less form.
Disambiguos	It is an operation by which ambiguous semantic cases are solved. In particular those cases which deal with homographs whose graphic form is the same but with a different meaning.
Lexicalization	To turn the unit into a string phrases consisting of two or three words that refer to a unitary meaning.
Cleaning the vocabulary	Words from empty or insignificant, such as the abbreviations techniques, proper names. articles, conjunctions are deleted.

We performed the analysis of the elementary context unit (e.c.u., like sentences or short texts characterized by the same patterns of keywords), carrying out some thematic clusters which were projected, as active variables, on the factorial plane through an analysis of multiple correspondences.

The analysis of the elementary context unit is based on a statistical study of the co-occurrence of words in the same e.c.u. The final product synthesizes the shared concerns in few significant thematic clusters as a contextual field of meanings (Reinert, 1995) that allow us to build up ‘a thread’ in the discourse. Lexical units (words or lemmas) are the result of a selection process aimed at creating a list of “key words”. Each cluster consists of a set of e.c.u. and is described through a set of keywords that, ranked according to the decreasing value of χ^2^, indicates that the typicality of each of them within the cluster is associated for semantic value. This allows to reflect on the meaning of individual words by reference to a number of e.c.u. analysing it in the context in which they are used. The meaning of a word is known only through its relations with the contexts, that is, through its distribution within portion of text (Greimas, 1983). Furthermore, according to the studies of Rommetveit (1998), Linell (2009), and Valsiner (2001), the language use and the semiotic systems can only be understood in their inter-relationship between culture, context and history; thus, the words and the clusters emerged have meanings only considering a network between individuals and environments.

Finally, the projection of clusters on the factorial plane allows to observe relationships (oppositions and neighbourhoods) between the issues emerged by interpreting the axes that bind them together.

## Results and Discussion

**Figure 1:**
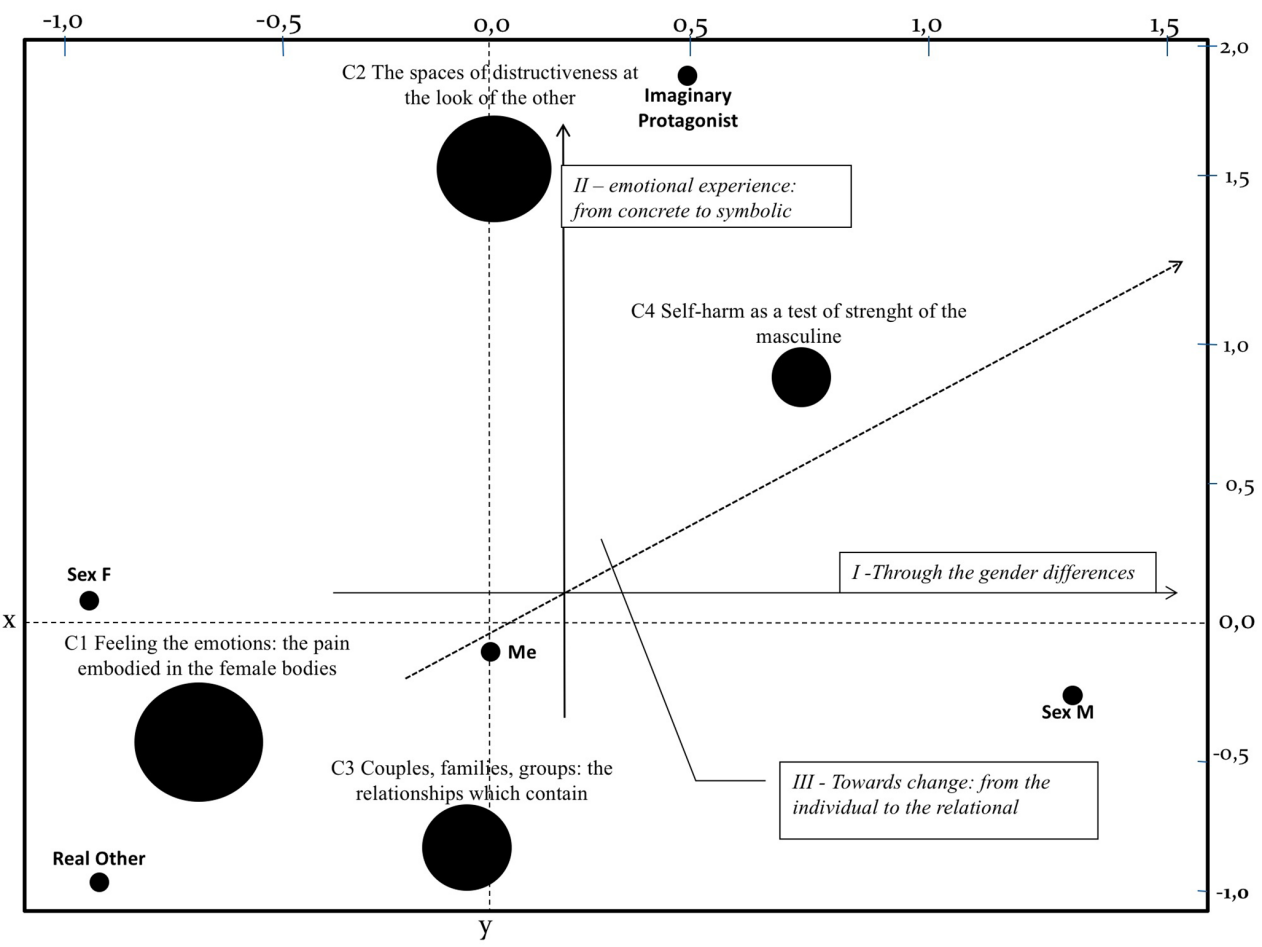
Factorial map of clusters.

We proceeded with the analysis of the clusters, according to their location on the factorial map (Figure 1) starting from the cluster that is statistically more significant (threshold value *p* = .05). For each cluster, to which an interpretative label has been assigned, the key words that mainly characterize it, based on the value of χ^2^, will be presented. Four thematic clusters emerged (Table 2).

**Table 2:** Description of Cluster

Percentage of cluster based on number of e.c.u.	Lemma that mainly characterize the cluster based on the value of χ^2^
Cluster 1 (composed by 97 e.c.u. on a total of 280 equal to 34.64%)	
feeling the emotions: the pain embodied in the female bodies	she (27.377), friends (22.302), meaning (16.576), people (10.082), hate (9.237), became (8.793), Fabiana (8.667), Marta (8.439), reply (8.439), know (7.948), injury (7.347), alone (6.771), hade (6,65), suicide (6.75), wrong (6.75), body (6.663), approach (6.345), continue (5.582), try (5.582), sense (5.464), bear (5.464), show (4.892), pain (4.785), seeing (4.654), feeling (4.222), help (3.918), provoke (3.918), sign (3.918).
Cluster 2 (composed by 83 e.c.u. on a total of 280, equal to 29.64%)	
the spaces of destructiveness at the look of the other	cry (23.771), come back (21.652), enter (18.84), professor (17.792), room (12.702), red (12.702), home (12.177), ended (10.158), quiet (10.158), white (10.158), look (8.984), happy (8.751), arrive (8.681), wound (8.632), door (8.609), call (8.609), mother (7.533), blood (7.476), love (6.606), punish (6.6.06), wish (6.606), immediately (6.558), wrist (5.776), leg (4.368), bathroom (4.207).
Cluster 3 (composed by 51 e.c.u. on a total of 280, equal to 18.21%)	
couples, families, groups: the relationships which contain	together (43.307), Alfredo (35.7649), month (34.026), tired (22.334), friendship (21.299), life (20.315), cousin (18.818), tell (16.985), take (16.258), class (14.831), family (14.166), strong (12.891), school (12.299), change (11.649), fear (11.649), round (10.779), useless (8.602), perfect (8.602), fun (8.602), cut (8.602), day (7.069), engage (5.817).
Cluster 4 (composed by 49 e.c.u. on a total of 280, equal to 17.5%)	
self-harm as a test of strength of the masculine	Drake (36.343), Michael (25.945), John (20.75), fire (19.99), big (18.328), high (15.79), physical (15.337), music (15.041), pleasure (12.322), unique (12.014), window (10.219), immense (10.219), protagonist (10.219), choose (10.219), own (7.091), last (7.091), listen (7.091), play (5.317). *Sex Male* (10.257).

*Note.* e.c.u = elementary context unit.

On the lower left quadrant of the factorial map we find cluster 1, named ‘*feeling the emotions: the pain embodied in the female bodies*’. This cultural context brings the issue of feminine into an emergency in the elaboration of the experience of self-harm. In fact, it is clear that the cluster incorporates many lemmas declined to the feminine (*she, friends, alone*, and the presence of female names of a person like *Fabiana* and *Marta*). The cluster aggregates also lemmas that lead back to a sensory level of self-harm practice (meaning, body, seeing, feeling, signs), telling us about the psychic suffering through a wounded female body (*wound*). References to the emotions of hatred, to the experiences of pain and loneliness, to suicide, as well as to the guilt (*wrong*) seem to shape female self-harm as particularly connected to a depressed mood, close to the drive of self-destruction, of death.

*A lonely and frustrated child mocked by everyone for the sole reason that she was not as beautiful as the others, she felt inferior. Every boy who passed only looked at her friends and never at her. Nobody was interested in knowing her and she felt so alone and wrong that she got to physically hurt herself* (Female, real story, protagonist other than herself).

*He decided to try to see if she could feel better too. He initially tried only with insignificant scratches. When he did he felt better, because he could vent all his anger. She vented on herself because she did not want to hurt the others* (Female, real story, protagonist herself).

Thus, the narratives that are aggregated in this cluster seem to offer us a representation of female self-harm as a marker of anguish, which passes through experiences of loneliness, suicidal ideas, feelings of inferiority and experiences of devaluation for one’s body. The emphasis is on the use of a sensorial-perceptive nature representation that starts from the body, from the colours, from the sensations.

On the upper right quadrant there is cluster 2, named ‘*the spaces of destructiveness at the look of the other’*. They join lemmas that refer to the spaces in which young usually self-harm, like the room or the bathroom of the house or school. At the same time, it seems that these places can be used in their psychic function to limit, as ‘borderline’ spaces where they can bring a request for help that moves from the body to the door. However, these boundaries, as real as psychic, expose to the other’s gaze, telling a possible unconscious need to be discovered by the mother, the professor (even the entries ‘enter’ and ‘get’ tell of someone who enters and discovers those injured). Moreover, in adolescence, interstitial spaces can be sought to bring many conflicting and secret contents, sometimes in an ambivalent form.

In addition, the cluster brings out different references to the size of the urgency (*immediately*), as well as to the injured parts of the body such as the wrist or the leg, or to colours ranging from clean white to red blood, all elements that connote the phenomenology of the self-harming act.

*Once inside, he took a cutter out of his pocket and made cuts on his wrist. He began to lose a lot of blood to the point that he fainted, a boy entered the bathroom and accidentally saw blood flowing out the door. He immediately called the janitors who brought the ambulance. The boy who had cut himself did not make it* (Male, invented story, protagonist of fantasy).

*... mother where he kept the razor blades, the boy took it, rolled his sleeves up and 1 2 3 4 5 cuts that made so much blood come out that the sink turned from white to all red the boy felt bad he stayed there crying for one hour thinking of that girl who one day before leaving called him best friend* (Male, real story, protagonist other than himself).

The second theme, therefore, represents the phenomenology of the self-harm symptom with reference to the concrete dimensions of the act (the wounding modalities, the injured parts) and the urgency of the symptom. The psychological operation that the adolescent is called to do in the invitation to narrate, is specular to that of the clinician, that is to move from the real to the symbolic, to go beyond the urgency of the concrete and beyond the factual reality, to access the psychic reality, to fantasy on facts. Through the narration we begin to talk, to name emotions, to attribute meaning to events, in a process of change in the relationship with the object, with the present and the past. Moreover, the spaces in which the symptom is brought are the liminal ones between the self and the other, psychic places in which to be watched, thresholds with psychic relational functions. Recalling Green (1991) and his theory of limit cases, we share that those who are self-harmers, beyond being and acting on the limit / edge, embody the elusive limit, they are the limit themselves.

Between the two quadrants at the top of the factorial map we find cluster 3, named ‘*couples, families, groups: the relationships which contain*’. This cultural context brings together lemmas that refer to sentimental relationships (*together, engagement*), *family* (*cousin*), as well as social (*friendship, class, scho*ol) that seem to act as good containers capable of accommodating disturbing (*fearful*) and non-disturbing (*fun*) contents; there is a search for a place where to store emotions, and it turns out that, besides the body, there is the other, the family and the peer group. At the same time, it is the cluster of relationships put to the test through provocative acts aiming at testing if the container can resist attacks (Ruggiero, 2007), as well as relational events that may presage self-destructiveness scenarios.

It is a cluster where words, that evoke the possibility of narrating experience (*telling*), also through references to temporality (*month, days*), are aggregated.

*Once there was nothing. There’s everything now, now there’s Alfredo, he’s not a prince, he’s all, he’s a friend, a body, he’s mine, he’s that person, even if you stab him, he’s not going, he’s heart, he’s body, life* (Female, real history, protagonist herself).

*A girl who had a life that was thought to be perfect... But then her cousin got sick ... The girl with her cousin did everything, they attended the same school and even the same class, they slept together and they had a loving family very tied up. In short, everything was perfect* (Female, real story, protagonist herself).

Offering the link to the relationship seems to be an important relapse, a possible answer, since, as Ladame (1983) points out, in cases of self-harm, we observe the presence of a primitive, archaic narcissism, which acts in an anti-relational direction, attacking the link. The urgency of the facts overwhelms the story, as well as the urgency of the symptom overwhelms the other by causing it to counter-acted and cancelling the space of suspension and think-ability. In this sense, then, the narrative of the standing bond seems to be an invitation to stay, to resist attacks, to tolerate frustration. Besides, also in the literature it has been shown how, on the clinical side, the relationship with such patients is difficult to establish and to maintain, often subject to interruptions and acts; where an insufficient basic narcissistic structure accentuates the dependence on the object, it is often the recognition of this dependence that triggers the use of violent action, in an extreme attempt to stem a threat of invasion and colonization by the object. In this sense, it is important to underline that intervention programs have been developed in schools that are sensitive to managing the relationship difficulties with such adolescents.

Finally, in the lower left quadrant cluster 4 is placed, called ‘*self-harm as a test of strength of the masculine*’. The cluster aggregates lemmas that refer to a grandiose dimension of oneself (*big, tall, physical*) and of what the protagonist is facing (*fire, immense*). This cultural unification represents male self-harming as physical resistance, proof of strength, far from how the feminine one is told. We can hypothesize the presence of a profound difficulty in representing himself as self-harmer in depressed and sad tones, preferring to narrate the adventures of a protagonist who wounds himself in war or in a duel, in a polarized dynamic on the maniacal side that denies a part of the self.

This dimension is approached through the use of imagination, the area of ​​‘as if’; in fact, the cluster is full of references to people far from reality, as the proper names allow us to understand (*Drake, Michael, John*), which instead seem to refer to heroes of novels, movies or video games.

*Drake raised his sword to the sky, while the fire deteriorated the little body that remained attached to him. He pointed the blade at himself and suddenly pierced the side of Drake* (Male, invented story).

*She started from the wrist, then the whole arm, then the calf, the thigh and the part she liked the most: the belly. How much she loved to see that hated belly that was being hurt. When she stopped she liked to touch the wounds and feel the blood flowing, she went to the bathroom and rinsed* (Female, real story, protagonist herself).

Finally, the representation of male self-harm seems completely overwhelmed by the practice of self-harm as a test of resistance to pain, where it seems difficult to describe oneself as suffering and where one prefers to use imaginary protagonists, vigorous heroes with whom it is reassuring to identify oneself. If self-harm in female representation is a marker of distress, the masculine one is a marker of physical endurance.

### Interpretation Using Factorial Axes

On the I factor, the horizontal one, we find the axis named ‘*through gender differences’*. This factor is organized along a continuum that opposes, as shown in the figure, on the one hand the self-harm to the feminine, using stories of real characters (positioning of the ‘other real’ variable), on the other hand, the cluster of male self-harm with its specific meanings and recourse to an invented story with a fictional character. In psychic terms, we interpret his polarization of genders on the physical graphic plane in the construction of a discourse on self-harming behaviours in scholastic contexts that is articulated in a semiotic trajectory on gender issues, a sense vector that gives us back the idea that a speech about self-harm as it emerges in these cultural contexts is something incarnate, of peculiar respect to the bodies and to their biological and cultural differences.

On the second factor, we find the vertical axis called ‘*emotional experience: from concrete to symbolic’*, which sees the opposition between a polarization based on the sensorial and physical dimensions of the emotions that are acted in self-harming conducts and in relational attacks, and on the other, an organization based on the representable experience of self-harm that, through the contexts in which it is acted, can access the plan of the imaginary and the symbolic. Therefore, on the one hand we find clusters 1 and 3 that are opposed to clusters 2 and 4 accompanied by the variable ‘imaginary protagonist’. On the psychic level, if from a pole there is the recognition of the concrete dimensions that pass through the body, on the other there is the possibility of access to a process of affective symbolization, which falls within spaces that become cultural contexts. It seems to prefigure a semiotic trajectory that pertains to the difficult process of emotional regulation: raw sensory elements, urgent and intense emotions such as anger and boredom need to be recognized, felt, tolerated, reclaimed, in a process that moves towards imagination and attribution of meaning.

On the III factor, named ‘*towards change: from the individual to the relationship’,* we find the clusters 1 and 4, based on the symptomatic declinations of the behaviour of self-harm, which are opposed to clusters 2 and 3, based on relational dimensions. This three-dimensional axis, both on the factorial and psychic level, grasping the depth of graphic space and internal space, allows the theme of self-harm to be harmonized, finding in the relationships the point of arrival for the various aspects involved in this process of signification. A third semiotic trajectory, therefore, appears as a relational one: it is in the link with the object that one can access the possibility of bringing a request for help in context. In conclusion, this vector of meaning opens up to possible interventions that investigate the area of relationships, which can sometimes prefigure self-harm scenarios.

## Conclusion

The present work aimed to explore the meanings about self-harm emerging within the school context, starting from the narratives shared by students with experiences of self-harm. Although some issues emerged from the analysis refer to the idea that self-harm is a behaviour strongly sensitive to gender differences, the study has highlighted that the area of ​​relationships is a dimension that unites adolescents with such behaviour, therefore both clinical and educational interventions are possible. Moreover, considering that self-harming behaviour acts as an emotional regulator, according to the literature (Gratz, 2007; Klonsky, 2007), being aware of using self-harm as a regulator of emotions is accompanied by the possibility of being able to express a request for help. So the centrality of the difficulties in regulating emotions in self-harm in adolescence highlights the need to work in order to develop programs that allow the recognition of emotions, so that adolescents can achieve effective regulation of the same, even in previous school years as a possible preventive measure.

In general, the work made it possible to observe that the phenomenon of self-harming circulates in school contexts and belongs to both the imaginary and the real level of adolescents, who respond positively when invited to tell it, as evidence of a desire to narrate as well to be seen and helped. Thus, in line with the literature on the subject (Bareiss, 2014; Hill & Dallos, 2012), narration is a functional tool for developing meaning and meaning-making processes in adolescents with conduct of self-harm; where the concrete plan of the act obscures the possibility of think disturbing emotions and memories, the narration of self-harm allows to access to a first symbolization and transformation of the emotion towards the representation of experience.

Through this study, therefore, we emphasize the importance of school as a valuable context of research and intervention to intercept young malaise. The quali-quantitative work allows reflections on specific needs, on possible areas of clinical intervention, offering guidelines for developing intervention protocols in school settings. In fact, prevention strategies can be developed for all adolescents as well as strategies for groups of adolescents at risk, or plans that include the training of teachers and parents, as suggested also in literature (Shapiro, 2008). Specifically, assuming the symptomatology of self-harm as strongly non-verbal and pre-verbal, it will be useful to develop safe environments for young adults with self-harming behaviours moderated by a psychologist, where is possible the welcoming, the naming of emotions, the encouraging of relationships, as well as an interpretation of behaviours.

Therefore, the trajectories of the discourse produced in the investigated cultural context can act as guidelines for possible trajectories of intervention within the same contexts. Thus, the issues that concern gender differences, the area of ​​emotional regulation, the difficulty in managing relationships can represent possible areas in which to draw up intervention plans and guidelines for work with both students and teachers in schools.

Among the limitations of the study we can include an unbalanced sampling by gender, even if the data analysis software allows to check this variability and to proceed to a qualitative study; the future aim is to extend the research sample.
